# Chlormethyl

**Published:** 1889-04

**Authors:** 


CHLORMETHYL, $C_2H_3Cl$., is being used extensively in Paris for local anesthesia since Vincent and Lailler produced that gas in greater quantities. A little tampon is blown at by a jet of the gas escaping from a syphon. This tampon is laid, with hard rubber pincers, on the spot to be operated upon. In a few seconds the skin turns white, and the anesthesia through the intense cold is produced. Neuralgias were treated that way by Debove. He had eight successes among ten cases of ischias, by daily applying the anesthesia to a spot where the nerve is near the skin. Sixty other neuralgias he cured permanently by this method.—*Wiener Klinische Wochenschrift.*

